# Adventures of a Tenth Century Medical Student

**Published:** 1895-01-19

**Authors:** 


### Adventures of a Tenth Century Medical Student.

In a former article we considered a case of surgical practice by an early mediæval bishop, illustrating, among other things, the diverse influences of religion upon medicine, the charity which urged the Christian to relieve his suffering neighbour counteracted by the mystic asceticism which forbade the priest to use his sanctified hands to benefit the vile body. The history of monastic medicine shows the like opposing tendencies. St. Benedict, founder of Western monasticism, declared that the care of the sick must be placed above all things; they are to be treated as representatives of Christ Himself. His followers do not venture to dispute this, but they observe that it is an extraordinary injunction, and they attempt to smother it beneath a lengthy commentary. Christians should look upon disease, not as an evil to be got rid of, but as did a certain pious hermit who, having long suffered from many chronic disorders, happened to pass a whole year in comparative health; "whereupon he mourned and lamented greatly, saying that God had forsaken him, and had not deigned to visit him that year." To the charity of St. Benedict they oppose the asceticism of St. Bernard, who went so far as to assert that it is unseemly for a Christian to take medicines. His pupil and successor, Abbot Fastred of Clairvaux, writes thus to a monk who complained that the monastic diet gave him headache and indigestion: "If you think a monk may take medicine you are utterly out of the way. Believe me, my father, I have often seen St. Bernard scruple to take oil and honey in his porridge to warm his stomach. And when I remonstrated, he used to reply—'Sickness is no excuse, for the holy fathers purposely built their monasteries in valleys and on damp slopes that the monks might be often ill, and being thus reminded of their death, be saved from carelessness.'" But on the whole charity predominated, and the monastic hospitals were not only, as Roger of Sicily, called them, "the solace of the poor, the harbours of the stranger and the destitute," but sometimes incited individual monks to seek some higher medical knowledge than could be found in the usual mediæval herbals and receipt books.

An interesting case is that of Richer, a monk of Rheims, in the tenth century, whose history of his own times, discovered about fifty years since, threw a welcome light on one of the darkest of mediæval epochs. Not finding sufficient opportunity for medical study at his monastery, Richer managed to communicate with a "secular clerk," Heribrand, who had set up as a teacher of medicine at Chartres. The result may be told in his own words.

"One morning (in 991) I met a horseman from Chartres, of whom I inquired his name, master, abode, and business. He replied that he was a messenger from Heribrand, a clerk of Chartres, to Richer, a monk of St. Remy. I said I was the man he wanted, and having kissed him, led him aside. Then he pulled out a letter inviting me to readings on the Aphorisms of Hippocrates. Greatly pleased, I hired a servant, and prepared to start at once, but my abbot gave me nothing more than a single pack horse. So I arrived without money, change of clothes, or other necessaries, at Orbais, a place famous for its hospitality. Here the lord Abbot D—— refreshed me with his conversation, and aided me by his munificence, and next day we set off for Meaux. We came, however, into a wood, where all kinds of misfortunes befel us. We lost the way, and made a detour of six leagues. The pack horse, before a fiery Bucephalus, now became lasier than an ass. Evening came on, the whole sky seemed descending upon us in rain, and we were still six miles from Meaux, when the strong Bucephalus fell down under my servant and expired, as if struck by lightning. How great our alarm and anxiety were those may judge who have been in similar plight. The boy, who had never travelled, lay exhausted on the ground, with the packages strewn around him. The storm increased our confusion; clouds and darkness encompassed us. Yet in these difficulties God failed not to inspire me with the following plan. I left the boy there with the baggage, told him what to say to strangers, and exhorted him to keep awake. Then I went on to Meaux with the man from Chartres. We found the bridge with difficulty, and then new perils came upon us, for it was so dilapidated that the townsfolk could hardly get across in daytime for their business. The man from Chartres, a bold and experienced fellow, sought for a boat, but in vain. Then he came back to the bridge, and, by God's help, putting his shield over the holes here, moving the planks there, running hither and thither, he got me and the horses across. It was pitch dark when I entered the monastery of St. Faro, where the brethren were still brewing a loving-cup; for it was a feast day with them, whence so late potations. I was welcomed like a brother, and refreshed with pleasant talk and plenty of food. But I sent the man from Chartres and the horses back to re-attempt the perils of the bridge, and look for the boy. He found him, after much wandering and shouting, in the second watch of the night, and brought him to the river. But, being afraid of the bridge, they sheltered in a hovel, where they found a bed but no food, though they had tasted nothing all day. How sleepless and anxious I remained they can judge who have watched for those dear to them. At daybreak they arrived famished. Food was given them, with fodder and straw for the horses. Then, leaving the boy with Abbot Augustine, I rode quickly to Chartres with the man, whence I sent the horses back for the boy. When he arrived, and I was freed from anxiety, I began zealously to study the Aphorisms of Hippocrates with Master Herehand, a man of much science and liberality. But since I learnt thence only the prognostic symptoms of disease, and this did not satisfy my desires, I begged him also to read with me the book called 'A Concordance of Hippocrates, Galen, and Soranus.' This he agreed to do, for he was a man of great experience in his art, and skilled in the principles of pharmaceutics, botanics, and chirurgics."

